# Characterization of the TRPV6 calcium channel-specific phenotype by RNA-seq in castration-resistant human prostate cancer cells

**DOI:** 10.3389/fgene.2023.1215645

**Published:** 2023-07-27

**Authors:** Clément Cordier, Aurélien Haustrate, Natalia Prevarskaya, V’yacheslav Lehen’kyi

**Affiliations:** Department of Biology, Laboratory of Cell Physiology, INSERM U1003, Laboratory of Excellence Ion Channel Science and Therapeutics, Faculty of Science and Technologies, University of Lille, Villeneuve d’Ascq, France

**Keywords:** RNA-seq analysis, expression profile, TRPV6 channel, castration-resistant prostate cancer, signaling pathway

## Abstract

**Background:** Transient receptor potential vanilloid subfamily member 6 (TRPV6), a highly calcium-selective channel, has been shown to play a significant role in calcium homeostasis and to participate both *in vitro* and *in vivo* in growth, cell survival, and drug resistance of prostate cancer. Its role and the corresponding calcium-dependent pathways were mainly studied in hormone-dependent human prostate cancer cell lines, often used as a model of early-stage prostate cancers. The goal of the present study was to describe the TRPV6-specific phenotype and signaling pathways it is involved in, using castration-resistant prostate cancer cell lines.

**Methods:** RNA sequencing (RNA-seq) was used to study the gene expression impacted by TRPV6 using PC3M^*trpv6−/−*^ *versus* PC3M^*trpv6+/+*^ and its derivative PC3M-luc-C6^*trpv6+/+*^ cell line in its native and TRPV6 overexpressed form. In addition to the whole-cell RNA sequencing, immunoblotting, quantitative PCR, and calcium imaging were used to validate *trpv6* gene status and functional consequences, in both *trpv6* ^*-/-*^ and TRPV6 overexpression cell lines.

**Results:** *trpv6* ^-/-^ status was validated using both immunoblotting and quantitative PCR, and the functional consequences of either *trpv6* gene deletion or TRPV6 overexpression were shown using calcium imaging. RNA-seq analysis demonstrated that the calcium channel TRPV6, being a crucial player of calcium signaling, significantly impacts the expression of genes involved in cancer progression, such as cell cycle regulation, chemotaxis, migration, invasion, apoptosis, ferroptosis as well as drug resistance, and extracellular matrix (ECM) re-organization.

**Conclusion:** Our data suggest that the *trpv6* gene is involved in and regulates multiple pathways related to tumor progression and drug resistance in castration-resistant prostate cancer cells.

## 1 Introduction

Prostate cancer (PCa) is one of the most common cancers in men worldwide, behind lung cancer in terms of incidence and fifth in terms of mortality. According to statistics from the World Cancer Observatory (http://gco.iarc.fr/), PCa affected 1,414,259 new cases worldwide in 2020, and more particularly in industrialized countries, with 3⁄4 of cases in men over 65 years of age (Sung et al., 2021). Initially, the tumors are androgen-dependent and respond to androgen deprivation therapy, resulting in retarded tumor growth. Nevertheless, the effectiveness of the latter is limited, and the tumors become insensitive to the androgens in 15% of cases, consequently qualifying as castration-resistant prostate cancers (CRPCs) (Fahmy et al., 2021). CRPCs are highly invasive and easily spread to other tissues such as lymph nodes and bones, leading to mortality in CRPC patients and partly explaining the decreased 5-year survival rate of PCa (Gandaglia et al., 2015).

Transient receptor potential vanilloid subfamily member 6 (TRPV6) is a membrane protein, a member of the transient receptor potential (TRP) ion channel family (Clapham et al., 2001). The TRPV6 cation channel is highly selective for Ca^2+^, with P_Ca_/P_Na_ values greater than 100, playing a crucial role in calcium homeostasis in various cell types (Bödding and Flockerzi, 2004), making this channel closely involved in intracellular Ca^2+^-related pathways and in bone mineralization (Lehen’kyi et al., 2007; Ma et al., 2020). Moreover, this channel is essential in intestinal calcium absorption (van Goor et al., 2017), the fertilization capacity of sperm (Weissgerber et al., 2012), in the exocrine functions of the pancreas (Masamune et al., 2020), and in the differentiation of keratinocytes (Lehen’kyi et al., 2011). It was also shown implicated in the pathophysiology of mutations, dysfunctions, and aberrant expression with the example of nephrolithiasis (Suzuki et al., 2008), chronic pancreatitis (Masamune et al., 2020; Zou et al., 2020), osteoporosis (Chen F. et al., 2014), and cancer progression (Lehen’kyi et al., 2012). TRPV6 overexpression in human malignancies is now established in thyroid, colon, breast, ovarian, and prostate carcinomas, being associated with tumor aggressiveness (Peng et al., 2001). As for PCa, it is highlighted by the total absence of this channel in healthy tissue.

Previous transcriptome analysis indicated that TRPV6 mRNAs were expressed in PCa and that channel expression correlates strongly with pathology stage and metastasis status (Peng et al., 2001). These studies suggest a potential role for the calcium channel TRPV6 in prostate cancer progression, where it is involved in the Orai1-mediated store operated calcium entry (SOCE) mechanism (Raphaël et al., 2014). Previous studies have reported that a knockdown of TRPV6 in hormone-sensitive LNCaP cells decreased the proliferation, accompanied by a decrease in the activation of the nuclear factor of activated T cells (NFAT), whereas in neuroendocrine tumors (Lehen’kyi et al., 2007; Skrzypski et al., 2016), it decreased the migration and invasion through matrix metalloproteinase 9 (MMP9) and cathepsin B (Kim et al., 2021) and decreased cell survival and resistance to chemotherapy (Raphaël et al., 2014). To date, most studies have investigated the role of TRPV6 in hormone-dependent human PCa cell lines, often used as a model for early-stage PCa. Our understanding of the extent to which the TRPV6 channel modulates the progression and intracellular signaling of CRPCs is very limited. Recently, the RNA-seq technology has been widely used in transcriptome analysis, study of intracellular pathways, and in the development of new targets.

The aim of the present study was to examine calcium-regulated signaling pathways in CRPCs. To explore the effects, we performed the complete gene expression profiling analysis, followed by Gene Ontology (GO) and Kyoto Encyclopedia of Genes and Genomes (KEGG) enrichment analysis, to highlight the biological roles of differentially expressed genes (DEGs) using PC-3M^*trpv6−/−*^ *versus* PC-3M^*trpv6+/+*^ and its derivative PC-3M-Luc-C6^*trpv6+/+*^ cell line in its native and TRPV6 overexpressed form.

## 2 Materials and methods

### 2.1 Cell culture

The PC-3M cell line was purchased from ATCC (Manassas, VA, United States). PC-3M-Luc-C6 cells were obtained from Caliper Life Sciences (Waltham, MA, United States). They were cultured in Roswell Park Memorial Institute 1640 (RPMI-1640) medium (Gibco-BRL, Cergy Pontoise, France) supplemented with 10% fetal bovine serum, kanamycin (100 μg/mL), and L-glutamine (2 mM). All cells were incubated under constant temperature and humidity conditions at 37°C and 5% CO_2_ atmosphere. The medium was changed two times a week, and cultures were split by treating the cells with 0.05% trypsin–EDTA for 5 min at 37°C before reaching confluence. To maintain *trpv6* ^*−/−*^ status of the cells, the antibiotic of selection puromycin at 0.1 μg/mL was added for the PC-3M^*trpv6−/−*^ cell line.

### 2.2 Plasmid and cell transfection

The plasmids vEF1ap-5′UTR-TRPV6_CMVp-mCherry vector and control vEF1ap-5′UTR_CMVp-mCherry vector (e-Zyvec, Loos, France) were obtained as previously described (Haustrate et al., 2023b). The cells were transfected using the Lipofectamine 3000 transfection reagent (Thermofisher, Massachusetts, United States) following the manufacturer’s instructions and were selected with the selective antibiotic G418 at 200 μg/mL.

### 2.3 RNA‐sequencing

Cells were plated in 25-cm^2^ dishes at 75% confluence, and total RNA was extracted and purified from cells using the NucleoSpin^®^ RNA Plus kit (Macherey-Nagel, Strasbourg, France) according to the manufacturer’s recommendations. Each RNA sample (three replicates per group) was validated for RNA integrity using an 18S/28S ratio. Then, 1 µg of total RNA from each sample (*n* = 10) was used for library preparation. Library preparation was realized following the manufacturer’s recommendations (Illumina Stranded mRNA Prep). Final samples pooled libraries prep were sequenced on ILLUMINA NovaSeq 6000 with SP-200 cartridge (2 × 800 millions of 100 bases reads), corresponding to 2 × 26 millions of reads per sample after demultiplexing. This work benefited from equipment and services from the iGenSeq core facility, at ICM (Paris, France).

### 2.4 Bioinformatics analysis

This work benefited from equipment and services from the iGenSeq core facility, at ICM (Paris, France). Bioinformatics analysis of the RNA-seq read data was performed at the IGenSeq Platform Brain Institute. Quality of raw data was evaluated with FastQC. Poor-quality sequences and adapters were trimmed or removed with DRAGEN, with default parameters, to retain only good-quality paired reads. The Illumina DRAGEN bio-IT Platform (v3.10.4) was used for mapping on the hg38 reference genome and quantification with *gencode v37* annotation gtf file. Library orientation, library composition, and coverage along transcripts were checked with Picard tools. Following analyses were conducted with R software. Data were normalized with DESeq2 bioconductor packages and DESeq2 workflow. Multiple hypothesis adjusted *p*-values were calculated using the Benjamini–Hochberg procedure to control the FDR. Finally, enrichment analysis was conducted using the clusterProfiler R package with gene set enrichment analysis, on the GO database and KEGG pathways.

### 2.5 SDS-PAGE and Western blotting

Total proteins were extracted from cells using RIPA buffer (10 mM Tris–HCl (pH 7.4), 150 mM NaCl, 5 mM EDTA, 1% Triton X-100, 1% sodium deoxycholate, 0.1% SDS, and protease inhibitors cocktail from Sigma). The lysates were centrifuged 15,000 × g at 4°C for 20 min and mixed with a La $\ddot{e}$ mmli buffer (125 mM Tris-HCl pH 6.8, 4% SDS, 5% β-mercaptoethanol, 20% glycerol, and 0.01% bromophenol blue) and boiled for 5 min at 95°C. Total protein samples were separated using 10% SDS-PAGE and transferred to a PVDF membrane with Power Blotter XL (Invitrogen, Massachusetts, United States). Membranes were incubated with anti-TRPV6 [1:500, (Haustrate et al., 2023a)] and anti-β-actin (Sigma-Aldrich, 1:1000, A5441). Immunocomplexes were visualized through the enhanced chemiluminescence method (Pierce Biotechnology Inc., Massachusetts, United States). Densitometric analysis was performed using a Bio-Rad image acquisition system (Bio-Rad Laboratories, CA, United States).

### 2.6 Quantitative reverse transcription-PCR (qRT-PCR)

RT-PCR experiments were performed as previously described (Lehen’kyi et al., 2007). Total RNA was isolated using the NucleoSpin^®^ RNA Plus kit (Macherey-Nagel, Strasbourg, France) according to the manufacturer’s recommendations. The quantitative real-time PCR of *TRPV6* and *GAPDH* mRNA transcripts was done using MESA GREEN qPCR MasterMix Plus for SYBR Assay (Eurogentec, Angers, France) on the BioRad CFX96 Real-Time PCR Detection System. The *GAPDH* gene was used as an endogenous control to normalize sample difference, degree of RNA degradation, and variability in RT efficiency. To quantify the results, the comparative threshold cycle method ∆∆Ct and Bio-Rad CFX Manager Software v2.0 were used. The primer sequences used for the qRT-PCR analysis were GAPDH: *5′-CTG​TTG​TGC​TCT​TGC​TGG​G-3′* and *5′-ACC​CAC​TCC​TCC​ACC​TTT​G-3’*; TRPV6: *5′- CCC​AAG​GAG​AAA​GGG​CTA​AT-3′* and *5′-TTG​GCA​GCT​AGA​AGG​AGA​GG-3’.*

### 2.7 Calcium imaging

Cells were plated onto glass coverslips and were loaded with 4 µM Fura-2 AM at 37°C for 45 min in the growth medium. Recordings were performed in HBSS (140 mM NaCl, 5 mM KCl, 2 mM MgCl_2_, 0.3 mM Na_2_HPO_3_, 0.4 mM KH_2_PO_4_, 4 mM NaHCO_3_, 5 mM glucose, and 10 mM HEPES adjusted to pH 7.4 with NaOH). CaCl_2_ was adjusted to 0 mM or 2 mM depending on the experiment. The coverslips were then placed in a chamber on the stage of the microscope. Fluorescence images of the cells were recorded using a video image analysis system (Quanticell). The Fura-2 fluorescence, at the emission wavelength of 510 nm, was recorded by exciting the probe alternatively at 340 and 380 nm.

### 2.8 Statistical analysis

Each experiment was performed at least three times, and all data were presented as the mean ± SEM. The independence between a qualitative and a quantitative variable was tested using *Student’s t-test* using the GraphPad Prism 9.5.0 program (GraphPad Software) to detect differences between groups. The results are expressed in terms of a statistical significance of *p* < 0.05. In the graphs, (*), (**), and (***) denote statistically significant differences with *p* < 0.05, *p* < 0.01, and *p* < 0.001, respectively.

## 3 Results

### 3.1 Validation of castration-resistant human prostate cancer PC3M and PC-3M-Luc-C6 cell line models

**FIGURE 1:**
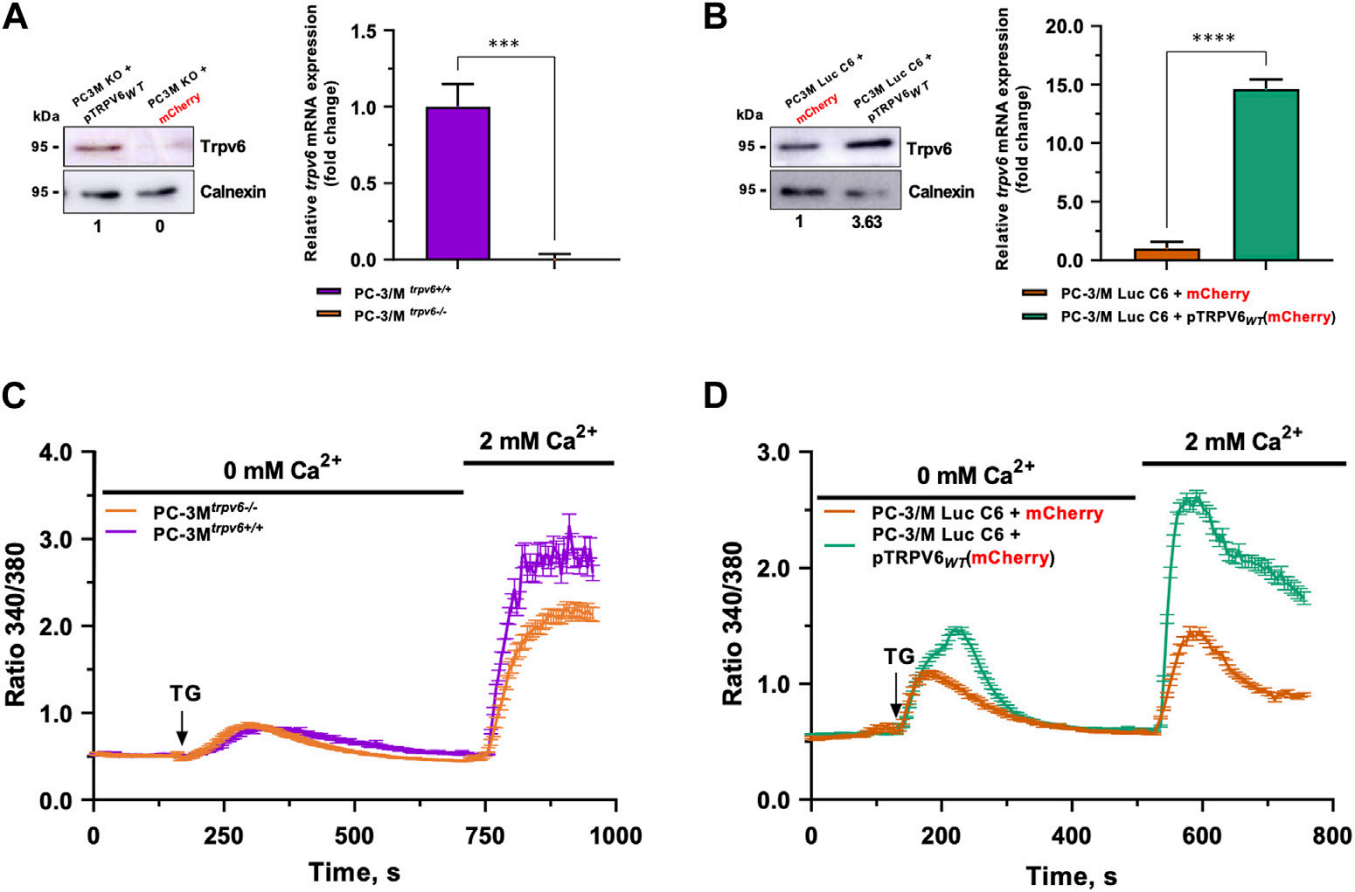
Validation of PC-3M and PC-3M-Luc-C6 cell line models and the store-operated capacitive calcium entry (SOCE) therein. The *trpv6* gene expression was analyzed by immunoblotting and real-time PCR in PC-3M **(A)** and PC-3M-Luc-C6 **(B)** cell lines. **(C)** SOCE in both PC3M^*trpv6+/+*^ and PC3M^*trpv6−/−*^ cell lines. **(D)** SOCE in both PC-3M-Luc-C6^*trpv6+/+*^ and PC-3M-Luc-C6^*trpv6+/+*^+ pTRPV6_WT_ cell lines.

The role of the TRPV6 expression in the castration-resistant cells was studied using the following cell lines: PC3M^*trpv6−/−*^ cells, PC3M^*trpv6+/+*^ cells, and their derivative PC3M-Luc-C6^*trpv6+/+*^ cell line in its native and TRPV6 overexpressed form. For this purpose, we realized a *trpv6* knockout in PC-3M and TRPV6 overexpression in PC-3M-Luc-C6 cell lines. PC-3M^*trpv6−/−*^ cells have no expression of TRPV6 protein as compared to the PC-3M^*trpv6+/+*^ cells, and thus no mRNA expression (Figure 1A). On the contrary, PC-3M-Luc-C6^*trpv6+/+*^+ pTRPV6_*WT*_ cells exhibited increased TRPV6 protein expression (3.63-fold change) and an increase in the mRNA expression (14-fold change) (Figure 1B) as compared to PC-3M-Luc-C6^*trpv6+/+*^ + mCherry cells, serving as the control.

TRPV6 was shown to be a channel playing an important role while amplifying SOCE in PCa cells, allowing the use of this mechanism to analyze the functionality of TRPV6 (Raphaël et al., 2014). Thapsigargin, which inhibits the sarcoendoplasmic reticulum calcium ATPase (SERCA) pump, is used at 1 µM to empty the calcium reserves and thus activate the store-operated channels (SOCs). Their activation will induce a translocation of the TRPV6 channels to the plasma membrane and thus amplify SOCE (Raphaël et al., 2014). SOCE was significantly decreased without TRPV6 expression in PC-3M^*trpv6−/−*^ cells (Figure 1C). On the other hand, in the model of TRPV6 overexpression in PC-3M-Luc-C6 cells, the SOCE increased significantly in both quantity and kinetics. Moreover, significant changes in the ER calcium content were noticed under these conditions (Figure 1D). Thus, both cellular models of differential TRPV6 expression demonstrate an altered calcium homeostasis according to the TRPV6 expression.

### 3.2 RNA sequencing analysis revealed a differential transcriptome profile in *trpv6* expression-modulated cell lines

**FIGURE 2:**
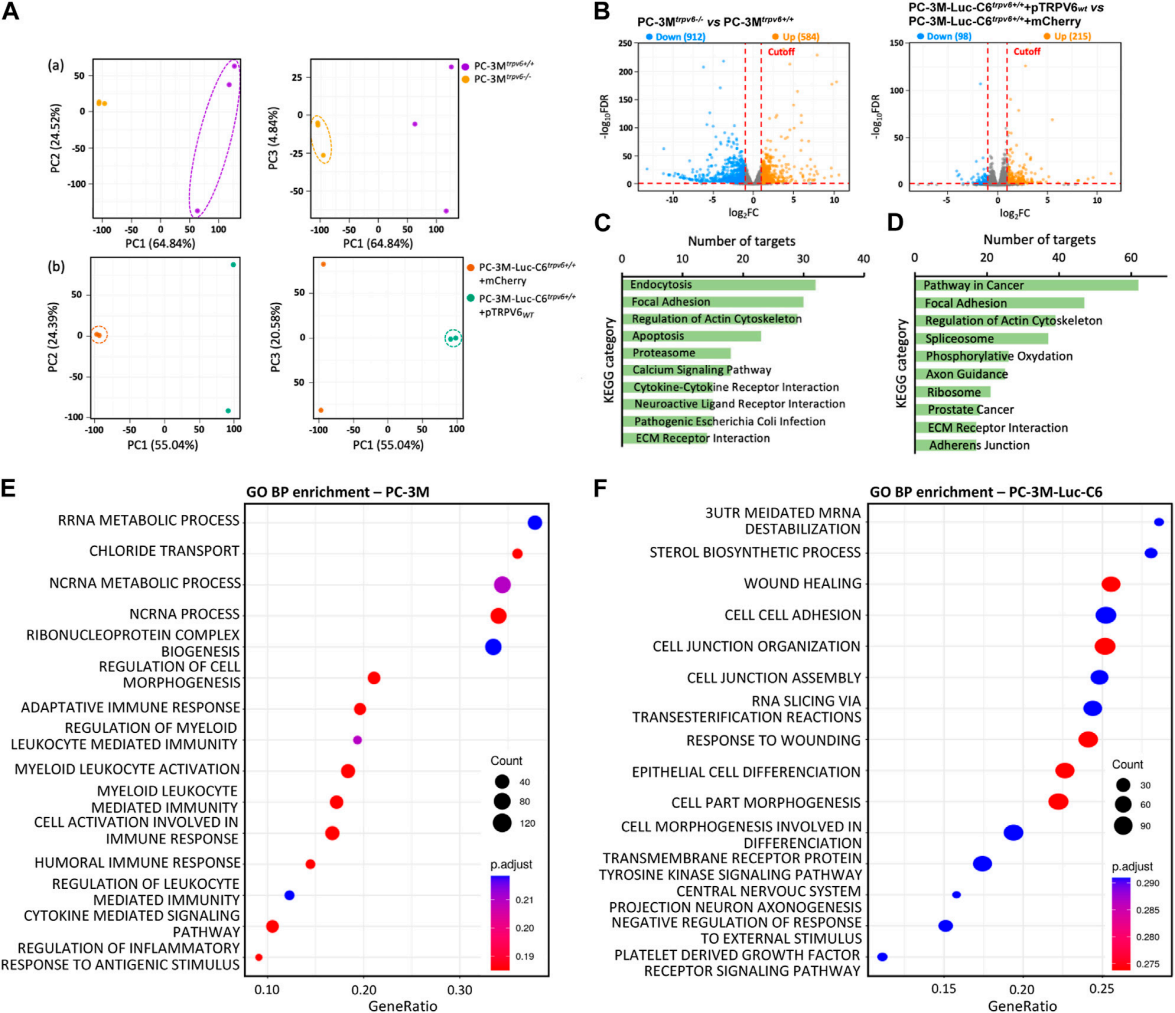
Transcriptional changes revealed by RNA-seq profiling of *trpv6* expression-modulated cell lines. **(A)** Principal component analysis showing the overall transcriptomic similarity between the (a) PC-3M and (b) PC-3M-Luc-C6 cell lines. **(B)** Volcano plots showing the differentially expressed genes (DEGs; Log2FC > 1 or < −1, false discovery rate adjusted *p*-value <0.05) in PCa cells. **(C,D)** Kyoto Encyclopedia of Genes and Genomes (KEGG) functional clustering of genes that were dysregulated for biological processes in PC-3M *versus* PC-3M-Luc-C6 cell lines, respectively. **(E,F)** Gene Ontology biological process (GO BP) functional enrichment analysis of DEGs in PC-3M *versus* PC-3M-Luc-C6 cell lines, respectively (terms of top 15 counts ranked by GeneRatio). The color and size of circles indicate the p-adjust and gene number, respectively.

RNA-seq was performed to analyze the transcriptomic features of the *trpv6* status and its functional implications within CRPC cells. The total number of the sequence reads ranges from 67 to 132 million in PC-3M cells and 67 to 87 million in PC-3M-Luc-C6 cells (Supplementary Table S1). Approximately, 99.7% of reads from each sample were aligned to the human genome (hg38). Gene expression was determined by the number of uniquely mapped reads to the specific gene and the total number of uniquely mapped reads in the sample. Principal component analysis of RNA-seq data (Figure 2A) showed that the transcriptome of PC-3M^*trpv6−/−*^ differed significantly from that of PC-3M^*trpv6+/+*^ cells, and also between PC-3M-Luc-C6^*trpv6+/+*^+mCherry and PC-3M-Luc-C6^*trpv6+/+*^+pTRPV6_*wt*_ cell lines.

Furthermore, the differential expression analysis was performed to determine transcriptomic changes induced by TRPV6-expression modulation in PCa cells. Figure 2B represents the volcano plots of the transcripts with |Log_2_FC| ≥ 1 (q value ≤0.05) in PC-3M^*trpv6−/−*^ cells compared to PC-3M^*trpv6+/+*^ cells as well as in PC-3M-Luc-C6^*trpv6+/+*^+pTRPV6_*wt*_ cells compared to PC-3M-Luc-C6^*trpv6+/+*^+mCherry cells. It becomes evident that TRPV6 expression induces an altered gene expression profile in these cells. A total of 1495 statistically significant DEGs were identified in PC-3M^*trpv6−/−*^ cells compared to PC-3M^*trpv6+/+*^ cells and 313 DEGs in PC-3M-Luc-C6^*trpv6+/+*^+pTRPV6_*wt*_ cells compared to PC-3M-Luc-C6^*trpv6+/+*^+mCherry cells. Approximately 61% of DEGs were downregulated in PC-3M^*trpv6−/−*^ cells, while 69% of DEGs were upregulated in PC-3M-Luc-C6^*trpv6+/+*^+pTRPV6_wt_ cells.

To characterize the biological roles of these DEGs in phenotype regulation, we used GO and KEGG pathway databases. Among the 1495 DEGs significantly regulated by TRPV6 expression in PC-3M cells, KEGG pathway analysis demonstrated the significantly affected categories in genes that were downregulated or upregulated in response to the *trpv6−/−*. The dysregulated genes were associated with endocytosis, focal adhesion, regulation of actin, apoptosis, calcium signaling, and ECM–receptor interaction (KEGG: 04144; KEGG: 04510; KEGG: 04810; KEGG: 04210; KEGG: 04020; KEGG: 04512) (Figure 2C). Among the 313 DEGs significantly regulated by TRPV6 expression in PC-3M-Luc-C6 cells, KEGG pathway analysis demonstrated the significantly affected categories of genes that were downregulated or upregulated in response to the overexpression of TRPV6. The dysregulated genes were associated with the PCa-related pathways such as focal adhesion, regulation of actin, spliceosome, and ECM–receptor interaction proteins (KEGG: 05200; KEGG: 04510; KEGG: 04810; KEGG: 03040; KEGG: 05215; KEGG: 04512) (Figure 2D). Furthermore, GO enrichment analysis showed that regulation of cell morphogenesis (GO: 0022604) and recruitment of the immune system were upregulated in PC-3M^*trpv6−/−*^ cells while the wound healing and cell–cell adhesion (GO: 0042060; GO: 0098609) in PC-3M-Luc-C6^*trpv6+/+*^+pTRPV6_*wt*_ (Figures 2E, F). Taken together, the data suggest that TRPV6 is closely involved in the PCa progression phenotype, including immune system escape and migratory capacity including cytoskeletal reorganization.

### 3.3 The most differentially expressed genes have key roles in cancer cell biology

**FIGURE 3:**
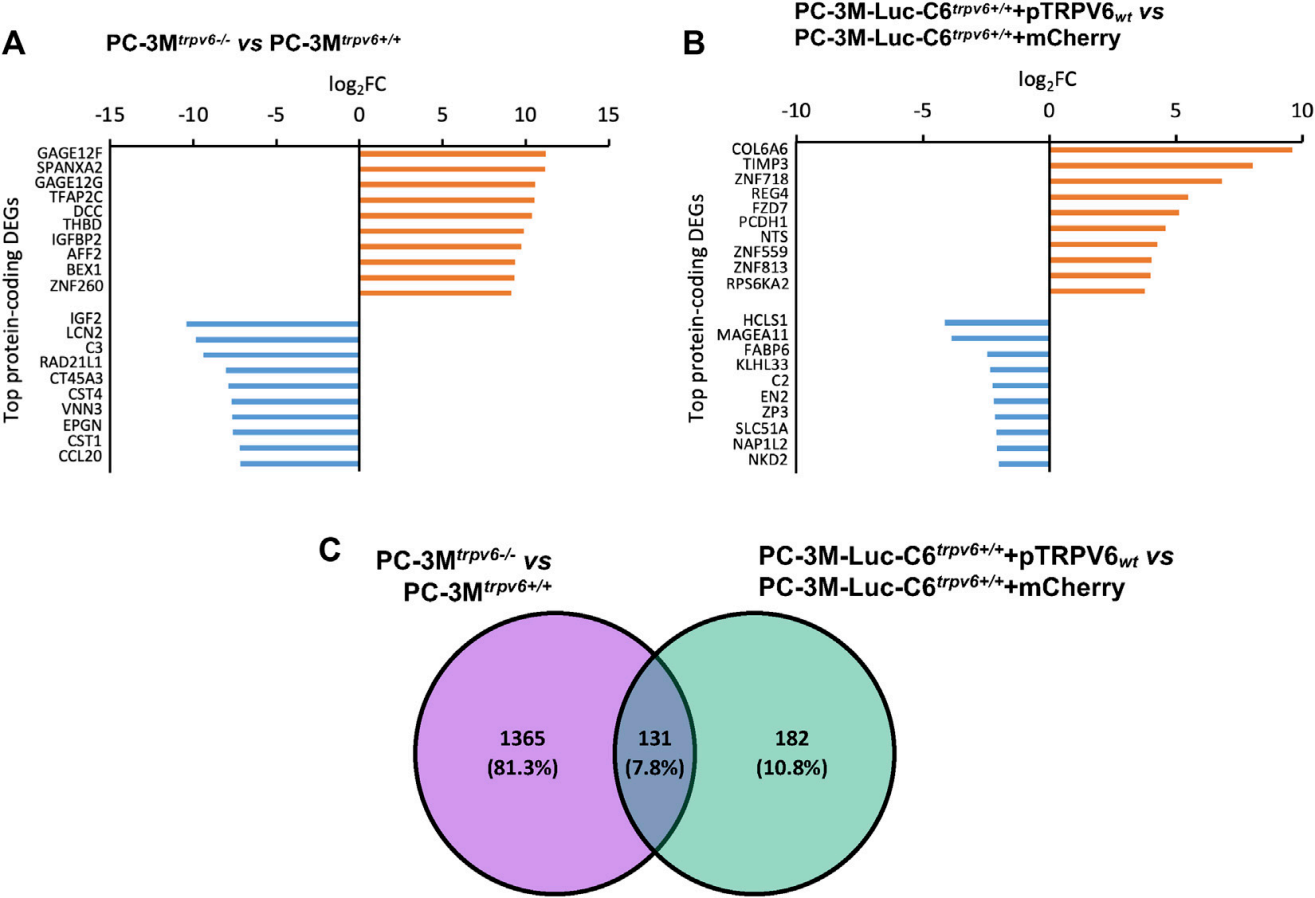
Differentially expressed genes (DEGs) in *trpv6* expression-modulated cell lines and their roles in cancer cell biology. **(A,B)** Top 10 protein-coding differentially expressed genes, upregulated (orange) and downregulated (blue), in PC-3M *versus* PC-3M-Luc-C6 cell lines, respectively. **(C)** Intersection of sets between the DEGs in PC-3M^*trpv6+/+*^ *versus* PC-3M-Luc-C6^*trpv6+/+*^+pTRPV6_wt_ cell lines.

A DEG sorting according to their adjusted *p*-value has been done. A complete list of top 10 protein-coding DEGs was obtained in our cell lines, as shown in Figures 3A, B with their log_2_FC. Major targets whose expression was increased by TRPV6 expression in the PC-3M^*trpv6+/+*^ cell line included *gage12f*, *spanxa2*, *gage12g*, *tfap2c*, *dcc*, *thbd*, *igfbp2*, *aff2*, *bex1*, and *znf260*. On the other hand, the targets, which were downregulated were *igf2*, *lcn2*, *c3*, *rad21l1*, *ct45a3*, *cst4*, *vnn3*, *epgn*, *cst1,* and *ccl20*. In the TRPV6 overexpression cell line, the major targets whose expression was increased by TRPV6 were *col6a6*, *timp3*, *znf718*, *reg4*, *fzd7*, *pcdh1*, *nts*, *zfn559*, *zfn813*, and *rps6ka2* and that suppressed was *hcls1*, *magea11*, *fabp6*, *klhl33*, *c2*, *en2*, *zp3*, *slc51a*, *nap1l2*, and *nkd2*. Among these gene targets, the majority of the genes highly affected by TRPV6 expression in PC-3M and PC-3M-Luc-C6 cell lines are those which are involved in cancer cell biology, notably in PCa, including tumor progression, bone lesions and metastasis, apoptosis and ferroptosis resistance-genes, and chemotherapy-resistant genes. While comparing the DEGs between PC-3M^*trpv6+/+*^ and PC-3M-Luc-C6^*trpv6+/+*^+pTRPV6_wt_ cells (Figure 3C), we noticed 131 shared genes, of which 64 DEGs are upregulated and 20 DEGs are downregulated in both cell lines. Among these common DEGs, a selection of these genes involved in cancer biology is shown in Table 1, including *abca13*, *ceacam6*, and *reg4* involved in acquisition of the chemotherapy-resistant phenotype; *cemip*, *cxcr4*, *fzd7*, *mrc2*, *pitpnm3*, and *clic2* playing a role in chemotactic cell migration, invasion, and metastasis; and *pcdh1* and *s100p* increasing PCa cell growth. The complete list of these DEGs is shown in Supplementary Data S1.

**TABLE 1:** Selected common DEGs upregulated or downregulated between PC-3M^*trpv6+/+*^ and PC-3M-Luc-C6^*trpv6+/+*^+pTRPV6_wt_ cell lines.

Gene symbol	Gene name	Role in cancer biology	Status with trpv6 (over)-expression	Refs
*abca13*	ATP-binding cassette sub-family A member 13	Cell membrane pump. Associated with conventional drug resistance and malignant progression in a variety of tumors	Upregulated	Hirschmann-Jax et al. (2004)
*ceacam6*	Carcinoembryonic antigen-related cell adhesion molecule 6	Cell surface glycoproteins. Involved in cancer progression, anti-apoptosis, resistance to therapeutic agents, and promotes cell invasion and metastasis	Upregulated	Rizeq et al. (2018)
*cemip*	Cell migration-inducing and hyaluronan-binding	Facilitates ferroptosis resistance during ECM detachment by promoting cystine uptake	Upregulated	Liu et al. (2022)
*cxcr4*	C-X-C chemokine receptor type 4	CXCL12 ligand promotes metastatic prostate cancer, in particular, in bone site	Upregulated	Akashi et al. (2008)
*fzd7*	Frizzled-7	WNT co-receptor Fzd7 activates WNT-β-catenin cascades and promotes PCa metastasis	Upregulated	Wang L. et al. (2022)
*mrc2*	Type 2 mannose receptor C	Regulates collagen remodeling and chemotactic cell migration through cooperation with matrix metalloproteinase 14 (MT1-MMP)	Upregulated	Kogianni et al. (2009)
*pcdh1*	Protocadherin-1	Induced Wnt signaling and permitted androgen-independent growth of hormone-sensitive cells	Upregulated	Terry et al. (2006)
*pitpnm3*	Membrane-associated phosphatidylinositol transfer protein 3	CC chemokine ligand 18 promotes cell migration and invasion and decreased apoptosis	Upregulated	Chen G. et al. (2014)
*reg4*	Regenerating family member 4	High expression correlated with tumor recurrence, metastasis, and therapy failure	Upregulated	Gu et al. (2005)
*s100p*	S100 calcium-binding protein P	Regulates calcium signal transduction and promotes prostate cancer progression by increasing cell growth	Upregulated	Basu et al. (2008)
*clic2*	Chloride intracellular channel protein 2	Plays preventive roles in malignant cell invasion and metastasis	Downregulated	Ozaki et al. (2022)

### 3.4 TRPV6 expression triggers genomic reprogramming of the aggressive phenotype in prostate cancer

**FIGURE 4:**
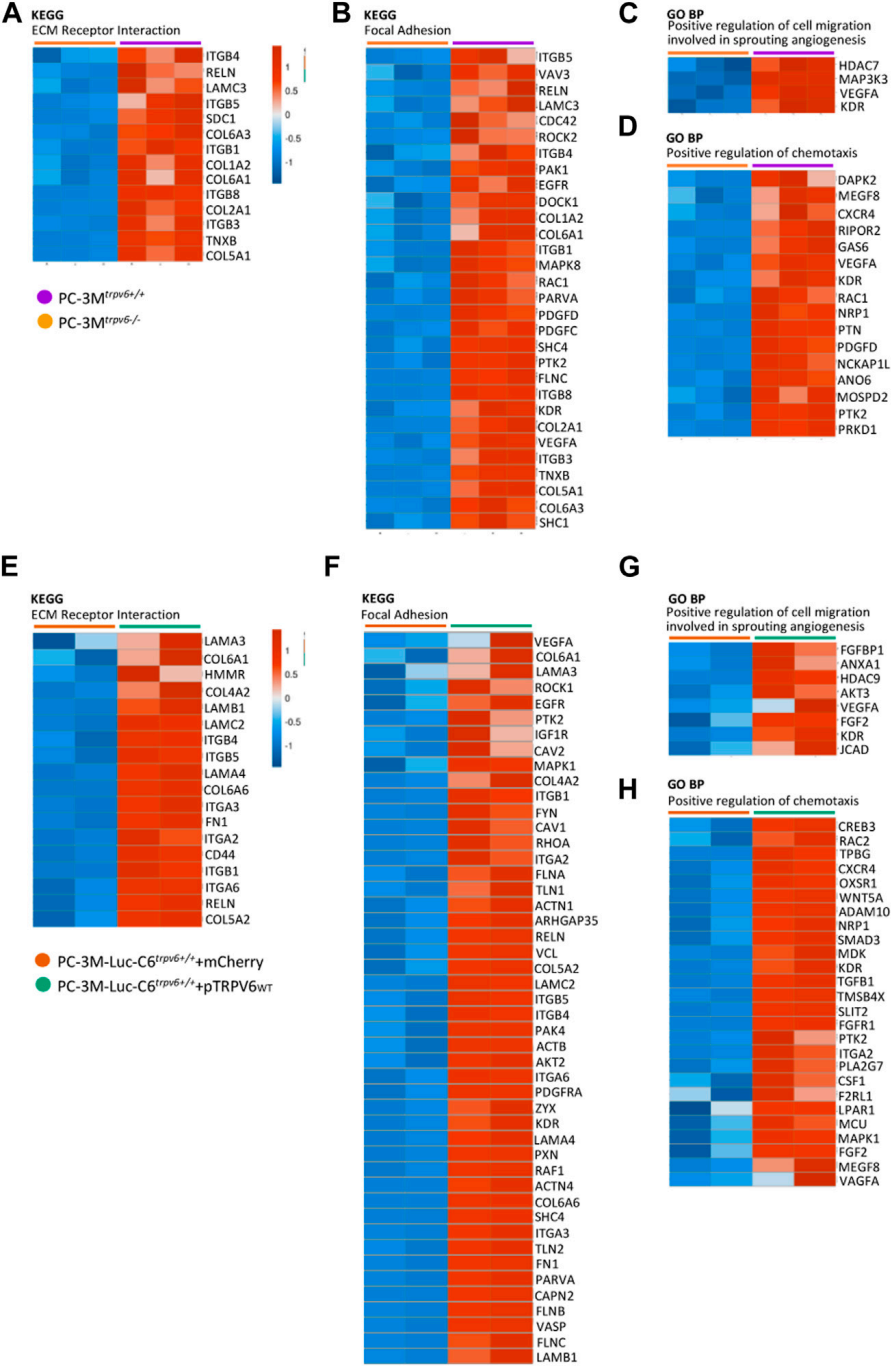
A heat map of the representative key canonical pathway genes in the aggressive phenotype in PC-3M and PC-3M-Luc-C6^*trpv6+/+*^ *.* Main genes involved in the ECM–receptor interaction **(A,E)**, focal adhesion **(B,F)**, positive regulation of cell migration which is involved in sprouting angiogenesis **(C,G)**, and positive regulation of chemotaxis signaling **(D,H)** are represented in the heat map if expressed in samples (RPKM >0), according to both the Kyoto Encyclopedia of Genes and Genomes (KEGG) and Gene Ontology biological process (GO BP) databases. Colors ranged from blue to red, corresponding from low to high expression levels, respectively (see scale bar).

The obtained data raised the question about the role of the TRPV6 protein in the acquisition of the invasive phenotype. Previous studies have suggested that TRPV6 could play a role in the acquisition of an aggressive phenotype, having a role in the migration, invasion, and metastasis of the pancreas, colorectal, breast, and the prostate cancer cells including osteoblastic lesions (Raphaël et al., 2014; Song et al., 2018; Arbabian et al., 2020; Ozaki et al., 2022). First, we studied both KEGG and GOBP pathways followed by the expression pattern from the RNA-seq data in order to visualize the molecular regulatory pathways that govern the acquisition of the aggressive phenotype in CRPCs (Figure 4). Genes reported as follows are from each canonical signaling pathway we obtained from both canonical signaling pathway KEGG and GOBP databases. Genes in each signaling pathway can be arbitrarily classified into two groups: increased or decreased expression patterns from PC-3M^*trpv6−/−*^ *versus* PC-3M^*trpv6+/+*^ and PC-3M-Luc-C6^*trpv6+/+*^+mCherry *versus* PC-3M-Luc-C6^*trpv6+/+*^+pTRPV6_*wt*_. It is known that the acquisition of the invasive phenotype is usually driven by factors such as remodeling of the cytoskeleton, forming the focal adhesion plaques, and close interaction with the ECM and chemotaxis, and we have successfully demonstrated these pathways. A majority of genes found in the KEGG ECM–receptor interaction, KEGG focal adhesion, and GOBP positive regulation of “chemotaxis” databases showed increased expression patterns in PC-3M^*trpv6+/+*^ and PC-3M-Luc-C6^*trpv6+/+*^+pTRPV6_*wt*_. These data suggested the direct role of TRPV6 in migration and invasion during the progression of PCa and participation of TRPV6 in the acquisition of the aggressive phenotype. Finally, the genes indicated in the GOBP database as “positive regulation of cell migration” and “involved in sprouting angiogenesis” are increased in these same conditions, indicating a possible involvement of this calcium channel in angiogenesis.

### 3.5 Impact on Ca^2+^ metabolism and differentially expressed genes related to other ion channels

**FIGURE 5:**
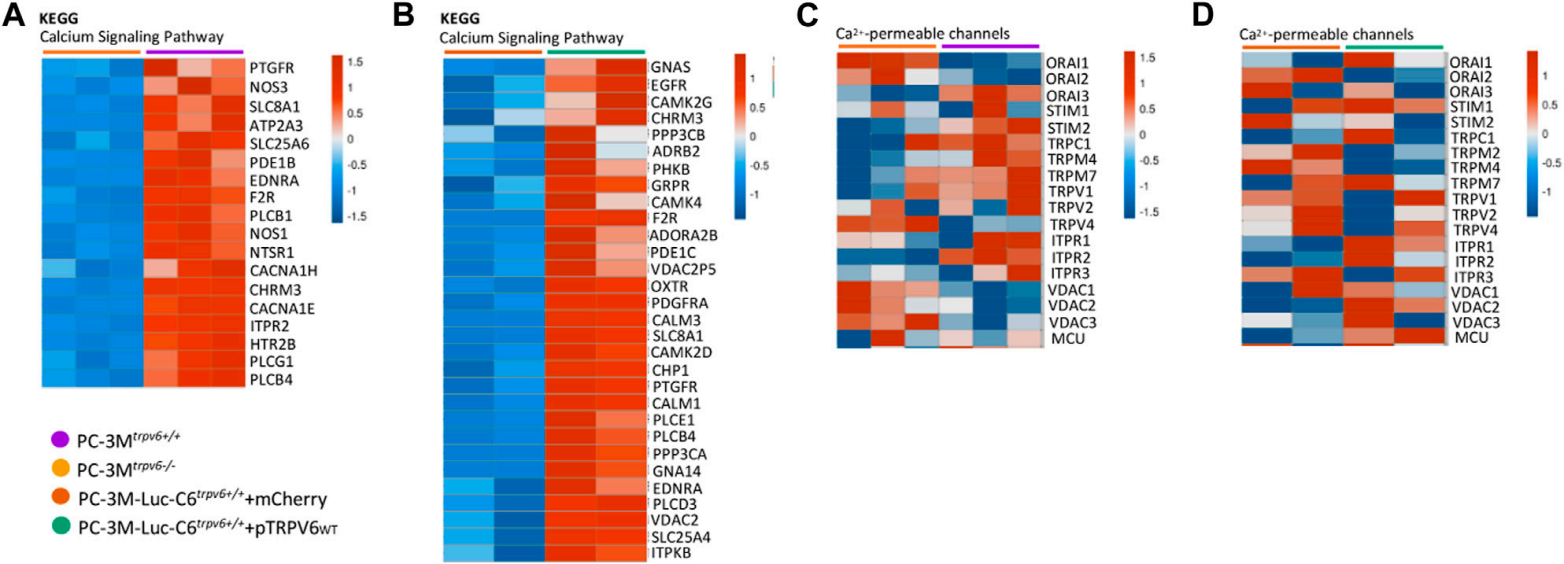
A heat map of the representative key canonical genes involved in calcium metabolism and also ion channels in PC-3M and PC-3M-Luc-C6^*trpv6+/+*^ cell lines. **(A,B)** Main genes involved in the “calcium signaling pathway” database according to Kyoto Encyclopedia of Genes and Genomes (KEGG). **(C,D)** Main ion channel genes in prostate cancer, realized with Morpheus, https://software.broadinstitute.org/morpheus. The genes were represented in the heat map if expressed in samples (RPKM >0). Colors range from blue to red, corresponding to low to high expression levels (see scale bar).

Calcium signaling is a central regulator of tumor cell initiation and progression, playing a role as a secondary messenger in numerous signaling pathways (Wu L. et al., 2021). This signaling is regulated by multiple channels and pumps, and we have already shown the impact of TRPV6 expression in Figure 1. This calcium modulation by TRPV6 led us to the study using the KEGG pathway database followed by the study of the expression pattern issued from the RNA-seq using mostly the genes from canonical Ca^2+^-mediated signaling pathways. As shown in Figures 5A, B, the majority of genes indicated in the KEGG “calcium signaling pathway” database showed increased expression patterns in PC-3M^*trpv6+/+*^ and PC-3M-Luc-C6^*trpv6+/+*^+pTRPV6_*wt*_, such as *ptgfr*, *egfr*, and *camk2d*, suggesting the functional role of the TRPV6 channel in Ca^2+^-mediated signaling pathways.

Furthermore, prostate cells express a number of plasma membrane ion channels, whose altered expression could be associated with phenotypic modifications and malignant transformation (Shapovalov et al., 2013). Knowing that Ca^2+^-permeable channels are involved in the establishment of tumor metastasis (Prevarskaya et al., 2011) and represent a group of diseases called oncochannelopathies (Prevarskaya et al., 2018), we examined the expression pattern using our RNA-seq data of different members of TRP, IP3Rs, and ORAI channel families. As shown by the heat map in Figures 5C, D, the TRPV6 expression was associated with alterations in the expression of several ion channel genes depending on the cell line. In PC-3M^*trpv6+/+*^, the expression of Orai1, TRPV4, and the VDAC channels decreases, in contrast to *orai3*, *stim2*, and *itpr2*. In PC-3M-Luc-C6^*trpv6+/+*^+pTRPV6_*wt*_, *trpm2* and *trpm7* are decreased as compared to *itpr1*, *vdac2*, and *mcu*. It is noteworthy that *orai2* was the only gene found to be consistently and significantly downregulated in both cell lines, PC-3M^*trpv6+/+*^ and PC-3M-Luc-C6^*trpv6+/+*^+pTRPV6_*wt*_. Taken together, these results indicate, on one hand, that TRPV6-mediated Ca^2+^ entry promotes Ca^2+^-mediated signaling pathways, which can promote tumor progression, thus confirming the data obtained previously. On the other hand, we have a transcriptomic profile of the calcium channels with altered expression and being specific to the cell lines with a decrease in the expression of *orai2* in the presence of TRPV6.

## 4 Discussion

The TRPV6 calcium channel is the most calcium-permeable channel of the TRP family, playing an important role in both physiology and pathophysiology, like cancer initiation and progression (Lehen’kyi et al., 2012). In the healthy prostate, the expression of the channel is absent but appears *de novo* and accompanies the progression of the malignancy having a role in cell survival. Our knowledge of the signaling pathways underlying TRPV6 *de novo* expression in CRPCs is still limited. To improve our understanding, we have used RNA-seq to characterize in-depth signaling pathways in CRPCs. The main aim of this study was to determine how the TRPV6 calcium channel in a human CRPC impacts the cell transcriptome.

We identified DEGs in CRPCs with the *trpv6*-modulated expression based on the gene expression profiles through the RNA-seq dataset, including PC-3M and PC-3M-Luc-C6 cell lines. Our results demonstrate broad transcriptional effects of TRPV6 between PC-3M^*trpv6+/+*^ and PC-3M-Luc-C6^*trpv6+/+*^+pTRPV6_*WT*_ cell lines that are highly cell-type-specific, including 584 upregulated genes and 912 downregulated genes in PC-3M^*trpv6−/−*^ and 215 upregulated genes and 98 downregulated genes in PC-3M-Luc-C6^*trpv6+/+*^+ pTRPV6_*WT*_. Strikingly, in both PCa cell lines, the overlap in DEGs was as little as only ∼8%.

Functional clustering of genes and functional enrichment analysis indicate that depending on a cell type, we have different TRPV6-modified biological processes and pathways, except for some pathways. For example, in both cell lines, where TRPV6 expression is altered, gene clusters associated with focal adhesion (KEGG: 04510), actin cytoskeleton regulation (KEGG: 04810), and ECM–receptor interaction (KEGG: 04512) are found in 10 functional clusters of genes that have been deregulated, indicating the transcriptional role of TRPV6 in cancer aggressiveness. Currently, only Kim et al. (2021) demonstrated a role of TRPV6 in the promotion of migration and invasion of PCa cells, without addressing a role on the remodeling of the cytoskeleton, studies which are mandatory to confirm their data at the phenotypic level. On the contrary, functional enrichment analysis shows cell-specific transcriptional effects.

The tumor microenvironment is increasingly involved in pathophysiological processes, including various actors such as the immune system (Hanahan and Weinberg, 2011). Moreover, such an interesting observation has been observed as the increase of the recruitment of the immune system cells in PC-3M^*trpv6−/−*^. Recently, a meta-analysis has revealed that patients with a strong immune cell infiltration and upregulated *tgf-β* and *wnt-β* signaling pathways had poor prognosis (Wu et al., 2022), suggesting a potential role of TRPV6, yet undiscovered. For PC-3M-Luc-C6^*trpv6+/+*^+ pTRPV6_*WT*_ cells, the functional enrichment analysis indicated a positive regulation in the pathways of wound healing, cell–cell adhesion, and cell junction organization, which is in agreement with the presumed function of TRPV6.

Overlapping DEGs play a particular role and can be divided into two groups. There are DEGs involved in cell survival including drug resistance, apoptosis resistance, and ferroptosis resistance involving *abca13* and *cemip*. The other DEGs are involved in the metastasis phenotype including chemotaxis, ECM remodeling, and activation of the Wnt signaling pathway involving *cxcr4*, *fzd7*, *mrc2*, *pcdh*, and *s100p*. Of them, ABCA13 belongs to the family of ATP-binding cassette transporters (ABC transporters) that can regulate the exchange of multiple compounds, including chemotherapeutic agents, and thus drug resistance represents a significant limit in the current therapeutic strategies. ABCA13 overexpression is associated with the malignant progression in PCa and glioblastoma, and it is also a marker of poor survival in metastatic ovarian serous carcinoma (Hirschmann-Jax et al., 2004; Nymoen et al., 2015; Dréan et al., 2018). CEMIP is a cell migration-inducing and hyaluronan-binding protein recently associated with poor prognosis when overexpressed in lymph node metastasis (Chen et al., 2022). Additionally, in LNCaP and PC-3 PCa cells, CEMIP inhibits ferroptosis, thereby facilitating cancer cell migration (Liu et al., 2022). CXCR4 is the C-X-C 4 chemokine receptor, which was shown to be implicated in aggressive phenotypes in various types of cancer such as breast and prostate cancer. Its ligand, CXCL12, was shown to be secreted by bone cells and promotes metastatic PCa (Akashi et al., 2008; Gandaglia et al., 2015). Recently, it has been shown that the simultaneous activation of CXCR4 and the histamine H1 receptor modulates calcium signaling and promotes migration (Park et al., 2023). In addition, the population of circulating tumor cells in PCa following radiotherapy was apparently more stable with the expression of CXCR4 (Klusa et al., 2023).

FZD7 is a Wnt co-receptor, and its expression in various cancers is associated with aberrant activation of the Wnt pathway promoting EMT, and thus metastatic development in prostate and pancreatic cancers (Wang L. et al., 2022; Zhang and Xu, 2022). In pan-cancer transcriptional and clinical data, MRC2 expression was significantly associated with the immune microenvironment such as immune cell infiltration, which is consistent with the functional enrichment analysis (Zhao et al., 2022). Moreover, it was demonstrated that MRC2 regulates cell collagen remodeling, and thus chemotactic cell migration (Kogianni et al., 2009). PCDH1 not only induced Wnt signaling activation in PCa (Terry et al., 2006) but also the progression of pancreatic ductal adenocarcinoma via NF-κB signaling (Ye et al., 2022). S100P is a part of the calciosome, which is the set of players regulating signaling mediated by calcium (Roderick and Cook, 2008), and more specifically, it has been shown to promote the progression of prostate cancer (Basu et al., 2008) and metastasis occurrence in colorectal cancer (Schmid et al., 2022). It is interesting to note that genes modulated by the expression of the TRPV6 channel are common to both the cell lines (see Supplementary Data S1), being essentially genes coding for proteins located at the plasma membrane. Thus, the identified genes may help in the development of new treatment strategies for advanced-stage prostate cancer, which is positive for the expression of the TRPV6 channel, and could constitute new diagnostic tools helping with the information regarding the aggressiveness of the cancer.

The expression profiles of key genes from canonical pathways like the ECM–receptor interaction (KEGG: 04512), focal adhesion cascade (KEGG: 04510), chemotaxis induction (GO: 0050927), and calcium signaling pathway (KEGG: 04020) further support the aforementioned conclusion. In fact, calcium is known to be an important player in the ECM assembly, the interaction between cell adhesion proteins and ECM proteins (Gopal et al., 2020), and also a main regulator of other pathways (Endo, 2006; Sánchez-González et al., 2010; Wu L. et al., 2021). For instance, the prostaglandin F2-alpha receptor (PTGFR), whose activity is mediated by calcium, has recently been shown to be able to participate in resistance to enzalutamide (Zheng et al., 2020) and may be associated with *in vivo* progression and the CRPC phenotype (Romanuik et al., 2010). It is already established that the epidermal growth factor receptor (EGFR) participates in tumorigenesis and progression of prostate cancer (DeHaan et al., 2009). However, recently, Nastały et al. (2020) indicated that the EGFR is a marker of bone metastasis obtained from CRPC patients. In the gene expression study, Zhang et al. (2016) identified calcium-/calmodulin-dependent protein kinase II delta (CAMK2D) as a DEG that may be associated with metastatic prostate cancer.

The expression pattern of key genes responsible for positive regulation of cell migration and involved in sprouting angiogenesis (GO: 0090050) has also been studied to evaluate other potential roles in the aggressive phenotype. Our data suggest that the upregulation of key gene targets under TRPV6 suppression or overexpression indicates that TRPV6 plays a crucial role via calcium signaling in angiogenesis, required for dissemination of prostate cancer cells to both adjacent and distant tissues. Indeed, several studies have reported that calcium signaling in cancer cells was linked to the angiogenic phenotype (Cui et al., 2017; Wang N. et al., 2022) via either the expression of hypoxia-inducible factor 1-alpha (HIF1ɑ) (Kim et al., 2015; Ma et al., 2018) or secretion of the vascular endothelial growth factor (VEGF), or inducing calcium oscillations in endothelial cells while binding to VEGF receptors (Wang N. et al., 2022; Li et al., 2022), or inducing cell proliferation, migration, and invasion needed to form new vessels.

TRP channels, known to be environmental sensors and permeable to calcium, modulate intracellular calcium concentrations and participate in the initiation and progression of cancers (Chen J. et al., 2014; Bai et al., 2023). Currently, only a few channels of the TRP family have been characterized as having a role in tumor angiogenesis, such as TRPV4, TRPV3, TRPM3, and TRPA1, demonstrated in lung carcinoma, renal cell carcinoma, cancer-associated fibroblast, and prostate cancers (Derouiche et al., 2017; Vancauwenberghe et al., 2017; Li et al., 2020; Kanugula et al., 2021; Li et al., 2022; Wei et al., 2022). No study reported a link yet between the TRPV6 channel and tumor angiogenesis, thus constituting a new line of research.

Ca^2+^ homeostasis is modulated within a network composed, in part, of different channels and transporters, such as SOC channels, TRP channels, IP3R channels, VDAC channels, and MCU channels, located at various cell membranes, plasma, or organelles (Clapham, 2007). In the presence of TRPV6, different cancer cell lines decrease the expression of Orai2, a calcium channel participating in SOCE. Nevertheless, the role of the Orai2 channel in cancer is still less studied, though it has been shown as an actor of cell cycle progression in breast cancer (Sanchez-Collado et al., 2021; Xu et al., 2021) and promoter of metastases in gastric cancer (Wu S. et al., 2021). No negative regulatory mechanisms between TRPV6 and Orai2 have been reported to date; therefore, further studies are needed to explore the molecular mechanisms.

Several studies have already published the transcriptional profile associated with a particular stage of PCa; however, the transcriptional profile of CRPCs associated with the expression of the TRPV6 calcium channel has never been studied. The same concerns other cancers. Several inhibitors directed against TRPV6 have been described, such as 2-APB (Kovacs et al., 2012), TH-1177 (Landowski et al., 2011), econazole (Neuberger et al., 2021), miconazole (Landowski et al., 2011), ruthenium red (Neuberger et al., 2021), and soricidin C-13 (Fu et al., 2017); however, all of them are not very specific. Our transcriptomic data suggest that anti-TRPV6 targeting could be used in combinatorial treatment to avoid any risk of migration to more or less distant secondary sites since metastasis formation is the main cause of mortality (Gandaglia et al., 2015). There would, therefore, be a need to develop more specific tools to target the TRPV6 channel in PCa, such as monoclonal antibodies (Bilusic et al., 2017).

The current study also has its limitations. The data obtained take into account only transcriptional effects which do not necessarily reflect protein-level modulations or phenotypic cell behavior, which may result from the post-transcriptional mechanisms. Investigating the effects of Ca^2+^-metabolism through the TRPV6 channel on the level of the proteome, the phospho-proteome, and the secretome of CRPC is certainly required. The data obtained from functional clustering of genes and functional enrichment analysis should be interpreted with caution as their significance is not always clear. For example, “endocytosis” (KEGG: 04144) is one of the KEGG pathways found being enriched by TRPV6 expression in PC-3M cells (32 DEGs associated with endocytosis are upregulated). Among these genes, some have an anti-endocytosis role, some have pro-endocytosis role, and some have a dual role. Another example is a “cell–cell adhesion” pathway (GO: 0098609) in the GO database found being enriched in TRPV6 overexpression of PC-3M-Luc-C6 cells, though not all of these genes have a role in adhesion. Thus, the functional effect of TRPV6 in the cell phenotype cannot be predicted by RNA-sequencing analysis alone. Nevertheless, these data provide insight into the cellular pathways that may be affected by a calcium-permeable TRPV6 channel in PCa cells. Further studies are needed to determine whether combined treatment strategies involving actual chemotherapy agents together with drugs that target this channel would significantly alter prostate tumor metastasis and drug resistance.

## 5 Conclusion

In summary, we identified several genes and pathways deregulated by altered TRPV6 expression using RNA sequencing and KEGG/GOBP databases. Our results reveal that TRPV6 appears to regulate the Ca^2+^ homeostasis and, thus, Ca^2+^ signaling pathways together with the expression of Ca^2+^-permeable channels and other pathways crucial for the development of aggressive and invasive phenotype in CRPCs. More precisely, the calcium channel TRPV6 alters the global transcriptomes of cancer cell lines and most notably the chemotaxis, migration, invasion, apoptosis, ferroptosis, drug resistance, and ECM organization pathways. Nevertheless, our research needs further experimental studies to validate our data and identify these molecular pathways at the protein level. TRPV6 targeting is likely to be prospective and can be used in new combined therapies to control the progression of castration-resistant prostate cancers.

## Data Availability

The original contributions presented in the study are publicly available. This data can be found here: https://www.ncbi.nlm.nih.gov/geo/query/acc.cgi?acc=GSE231352. Accession number: GSE231352.
