# Supplementary material for: Characterization of the TRPV6 calcium channel-specific phenotype by RNA-seq in castration-resistant human prostate cancer cells
Source: Front Genet. 2023 Jul 27;14:1215645. doi: 10.3389/fgene.2023.1215645 (PMC10415680; doi:10.3389/fgene.2023.1215645)
Supplement: Supplementary file 1 [file Table1.docx]

Supplementary Table 1

Data analysis of RNA-seq in PC-3M and PC-3M-Luc-C6 cell lines.

| **Cell Lines** | **Sample ID** | **Total reads** | **Reads mapped** | **% of reads mapped** |
| --- | --- | --- | --- | --- |
| PC-3M*^trpv6+/+^* | PC3M_wt_1 | 131,674,480 | 131,387,791 | 99.782274439208 |
|  | PC3M_wt_2 | 78,365,714 | 78,197,353 | 99.7851598723391 |
|  | PC3M_wt_3 | 79,413,114 | 79,219,372 | 99.7560327378675 |
| PC-3M*^trpv6-/-^* | PC3M_KO_1 | 67,293,726 | 67,112,962 | 99.7313806044861 |
|  | PC3M_KO_2 | 79,697,896 | 79,461,399 | 99.703258163804 |
|  | PC3M_KO_3 | 79,984,196 | 79,768,184 | 99.7299316479971 |
| PC-3M-Luc-C6*^trpv6+/+^*  +mCherry | PC3M_Luc_C6_1 | 87,040,868 | 86,825,400 | 99.7524519171845 |
|  | PC3M_Luc_C6_2 | 75,855,194 | 75,691,749 | 99.7845302458787 |
| PC-3M-Luc-C6*^trpv6+/+^*  +pTRPV6*_wt_* | PC3M_Luc_C6_pwt_1 | 66,949,272 | 66,792,155 | 99.765319330134 |
|  | PC3M_Luc_C6_pwt_2 | 76,541,274 | 76,357,777 | 99.7602639851539 |
